# Volatile Organic Compound Profiling of Commercial Poi Products in Fresh and Aged States Using Comprehensive Two-Dimensional Gas Chromatography

**DOI:** 10.1021/acsomega.5c01820

**Published:** 2025-05-15

**Authors:** Sarah C. Foster, Cynthia Cheung, Laura Tipton, Jonathan D. Baker, Kahoalii K. Keahi-Wood, Katelynn A. Perrault Uptmor

**Affiliations:** † Nontargeted Separations Laboratory, Chemistry Department, 8604 William & Mary, Williamsburg 23185, Virginia, United States; ‡ Laboratory of Forensic and Bioanalytical Chemistry/Forensic Sciences Unit, 3941 Chaminade University of Honolulu, Honolulu 96816, Hawaii, United States; § School of Natural Sciences and Mathematics, Chaminade University of Honolulu, Honolulu 96816, Hawaii, United States; ∥ Departments of Biology and Mathematics & Statistics, James Madison University, Harrisonburg 22807, Virginia, United States

## Abstract

Taro (Colocasia esculenta L.) is a plant originating from Southeast Asia now prevalent throughout the Pacific Islands. Growing taro was and remains an important facet of the Hawaiian culture. Poi is a food product prepared from steamed taro that has been macerated and allowed to ferment. While poi is commonly prepared at home, it can also be found commercially from select Hawaiian brands. The general fermentation process of poi produces volatile organic compounds (VOCs) such as alcohols, aldehydes, ketones, organic acids, esters, and heterocycles, among others, through microbial and enzymatic reactions. The VOCs found in poi are a class of compounds traditionally analyzed through gas chromatography (GC)–mass spectrometry (GC–MS) but have not previously been characterized in the literature. This study aimed to identify a core VOC profile of commercial poi products to better understand sample composition. Changes in fresh to aged poi were observed as well as its brand specific profiles. Analysis was conducted using comprehensive two-dimensional GC-quadrupole mass spectrometry with flame ionization detection (GC×GC–qMS). Samples were prepared according to package instructions, extracted via headspace SPME Arrow, and analyzed in replicates of 10. A total of 56 analytes were identified across three brands of commercial poi, ranging from 11 different compound classes. All brands showed a clear distinction between fresh and aged samples. Fresh samples across all brands contained 4,4-dimethoxy-3-methylbutan-2-one while aged samples all contained 2,5-dimethylfuran. Within the fresh state, the VOC profiles from each brand appeared to be distinct from one another. Taro brand poi was the least similar to Hanalei brand poi and He Mea Ono brand poi. He Mea Ono and Hanalei had some overlap in VOCs, but a substantial portion of their VOC profile was unique to the individual brand. In the aged state, there was a significant overlap among all three brands, which indicates that fermentation caused the VOC profiles to become more similar to one another. Visual distinctions in chromatographic plots were supplemented by principal component analysis and box plots, identifying distinguishing compounds. Investigating the profile of poi via GC×GC–qMS enhanced our understanding of its unique qualities, historical context, and contemporary use.

## Graphical abstract

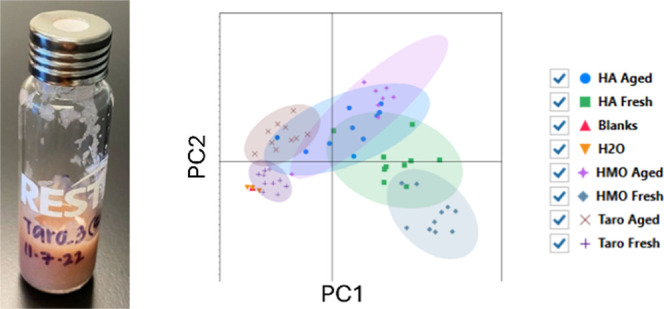

## Introduction

Taro (Colocasia esculenta L.) is a tropical corm with origins in southeast Asia and today is found in tropical and subtropical regions around the world. The growth and cultivation of taro have historical roots in the Pacific Islands, especially ‘Hawai‘i. Due to its humid climate, Hawai‘i offers ideal conditions for producing taro.^1^ Apart from its agricultural prominence, taro is significant in its medicinal and nutritional applications; it is often fed to infants with indigestion and is a significant source of probiotics, vitamins, and minerals.^2,3^ Poi is a food product made from the taro corm, the underground storage organ of the plant, that has been steamed, macerated, and fermented.^2^ Although poi is still commonly prepared within households, there are also several brands that are available commercially and can be purchased at grocery stores.

The flavor of food products is associated with a broad array of volatile organic compounds (VOCs) originating from their ingredients. The biological process of fermentation also contributes to this VOC profile.^4^ The mixture of VOCs from poi and its fermentation can be examined through analytical techniques, such as gas chromatography (GC). Traditional GC techniques separate analytes within a capillary column coated with a stationary phase, which allows analytes to separate based on their chemical affinity for the column coating. More complex samples benefit from the higher resolution and separation capacity of comprehensive two-dimensional gas chromatography (GC×GC), which is becoming common for the analysis of many food and beverage industries such as beer, wine, honey, olive oil, and more.^5^ In GC×GC, separation is performed using two columns with different stationary phases separated by a modulator, which allows separation based on two independent retention mechanisms.^5^ This improves the identification of components in complex samples as it reduces uncertainty resulting from the coelution of compounds with similar properties.^6^ Further analysis can be achieved through combining GC×GC with mass spectrometry detection.^7^ Quadrupole mass spectrometry (qMS) data (albeit slower than time-of-flight) has been previously shown to be capable of sufficient data rate for integration and is often combined with a secondary detector such as a flame ionization detector (FID).^8^ While the qMS/FID dual detection approach is common in petroleum industries, it has also been demonstrated previously on forensic samples^8^ and other Hawaiian food products.^9^ The application of GC×GC in food industries has become increasingly relevant as studies focus on the link between aroma and food consumption, as well as the use of aroma to detect food origin, quality, adulteration, shelf life, and more.^6^

Due to sufficient detector linearity and acquisition rate for analysis previously demonstrated,^8^ this study focused on applying qMS stream data from a GC×GC instrument equipped with reverse fill/flush modulation for the differentiation of poi sample types. GC × GC-qMS has been used in prior aroma profiling studies, including those related to fermentation.^10,11^ Several studies using GC and GC–MS have been conducted on fresh taro corm to determine its volatile constituents.^12–14^ Further, Huang et al. utilized high-performance liquid chromatography to quantify changes in organic acids and simple sugars in fermented poi as well as microbiological techniques to identify the bacteria responsible for the fermentation process.^15^ However, there is limited research pertaining to the aroma profile of poi specifically, and no study has ever employed the nontargeted power of GC×GC for this application.

This study aimed to observe changes in fresh to aged commercially purchased poi using comprehensive two-dimensional GC-quadrupole mass spectrometry with flame ionization detection (GC×GC–qMS). The purpose of this investigation was separated into three goals to establish: (1) a core VOC profile of poi, (2) differences in VOC profiles of three commercial poi products, and (3) changes to the VOC profile that occur during the fermentation of poi products. Providing a more detailed profile of the composition of poi products will serve to improve the understanding of their flavor and aroma and its historical context through cross-referencing the chemical profile against traditional uses, as well as better understand its use as a contemporary food product.

## Results and Discussion

After data processing, a total of 56 analytes were identified across the three brands of commercial poi ranging from 11 different compound classes. A complete list of analytes is available in Table S1 within the Supporting Information, along with example chromatograms from the analysis.

### Core VOC Profile of Poi

The core VOC profile of poi is the set of analytes present in all three commercial brands of poi. A total of 24 components were identified as present in Hanalei brand poi, He Mea Ono brand poi, and Taro brand poi. Seven components were consistently identified between fresh and fermented samples across all brands including 1-pentanol, 2-methylpropan-1-ol, 3-hydroxybutan-2-one, aminourea, acetic acid, pentane, and propanedioic acid. Excluding aminourea, each of the identified components have been documented as or are related to established taro VOCs.^13,14^ Aminourea, although not typically found in food products, could be a byproduct of agricultural treatment as it could be related to fertilization, soil treatment, or industrial processing.^16^ There were 17 VOCs that were identified consistently across every brand that appeared in either the fresh or aged states or both (Table 1).

**Table 1:** List of Analytes and Their Compound Class Found in at Least One Aging State Across Every Brand^a^

components	HA fresh	HA aged	HMO fresh	HMO aged	taro fresh	taro aged	class
1-prop-2-ynoxypropan-2-ol*	x	x	x	x		x	alcohol
2-methylbutanal	x			x	x	x	aldehyde
acetaldehyde	x		x	x	x	x	aldehyde
penta-1,4-diene*	x		x			x	alkene
2,5-dimethylfuran		x		x		x	heteroaromatic
2,2,3,3-tetramethyloxirane*	x		x	x	x		epoxide
2-methylpropyl acetate	x	x	x	x		x	carboxylic acid ester
3-methylbutyl acetate	x	x	x	x		x	ester
butyl acetate	x	x	x	x	x		carboxylic acid ester
ethyl acetate	x	x	x	x		x	carboxylic acid ester
ethyl propanoate	x		x	x	x	x	carboxylic acid ester
formyl acetate*		x	x	x		x	carboxylic acid ester
methyl acetate	x	x	x	x		x	ester
n-propyl acetate	x	x	x	x		x	ester
2-hydroxypentan-3-one	x	x	x	x	x		ketone
4,4-dimethoxy-3-methylbutan-2-one*	x		x		x		ketone
urea		x		x	x	x	nitrogen-containing

a Highlighted with an asterisk* have no documented natural sources and may be associated with processing, packaging, and/or storage of the food product. “HA” refers to Hanalei brand poi, “HMO” refers to He Mea Ono brand poi, and “Taro” refers to Taro brand poi.

Carbonyl compounds, such as aldehydes, esters, and ketones, are naturally occurring and contribute to the aromatic profile of poi in its “fresh” state. The identified aldehydes, 2-methylbutanal and acetaldehyde, can form as a product from the fermentation of sugars and catabolism of amino acids by yeast.^12^ Esters and ketones also form as a byproduct of bacterial and yeast metabolism, accounting for compounds present in the “aged” state as well as the “fresh” state. Carbonyl compounds generally contribute fruity, floral, nutty, and sweet odors to a food.^17^ For example, in a study examining fermented beverages, the “fruity” flavor of ryazhenka (fermented milk) and mahewu (fermented maize meal) is attributed to an abundance of ketones and esters, respectively.^18^

One analyte, 2,5-dimethylfuran, was present in all three brands in the “aged” state only. Furans have been documented in taro corm cooked at high temperatures as products of sugar degradation.^13^ In addition, 2,5-dimethylfuran forms in carbohydrate-rich fermentation conditions; since taro corm consists of mostly water and starch, its common presence is justified.^12^

Several analytes were identified but are not naturally abundant: 1-prop-2-ynoxypropan-2-ol, penta-1,4-diene, 2,2,3,3-tetramethyloxirane, formyl acetate, and 4,4-dimethoxy-3-methylbutan-2-one. There are several possible mechanisms for their detection. First, it is possible that these analytes are present in the samples but that they have not been previously identified in other studies. They could also potentially occur as products of side reactions between other poi VOCs, especially since GC×GC–qMS has several zones with high temperatures. They could also be attributed to packaging contamination, incorporation during processing procedures, or other introductions during the manufacturing and sales pipeline. Further investigation of unused packaging or analysis at different points in processing would be necessary to confirm the source of these specific analytes. Since packaging was not retained in this study, it was not analyzed retroactively; however, future studies could be designed to obtain poi products before packaging, after packaging, and after storage over a period of time. Because they were detected consistently in samples at relatively abundant levels as well as completely absent in water and method blank samples, they were left within the final peak table for documentation.

Overall, the core VOC profile of poi in this study consists of 24 compounds belonging to 10 different compound classes. When referencing aroma indicators from safety data sheets and flavor and fragrance databases,^19–21^ the general aromatic profile of poi based on the analytes identified across all three brands, regardless of aging status, was consistently found to be sweet, fruity, and nutty. Describing the flavor or aroma of complex food products, especially those with fermentation processes, can be challenging for consumers. Due to flavor being the most important feature in determining a consumer’s product preference,^4^ chemical analysis provides some objective basis for understanding the odor of poi and other products. Since odor is a physical property based on chemical presence, odor profiling contributes valuable foundational data toward describing the odor of specific foods with complex composition.

### VOC Profiles of Commercial Poi Products

While each of the three commercial poi brands shared a core set of VOCs, they also contained unique VOCs that could help differentiate one brand from another. These similarities and differences are highlighted in Figure 1. Hanalei brand poi contained 46 total analytes, 12 of which were found only in the “fresh” state, 11 of which were found only in the “aged” state, and 23 of which were found in both the fresh and aged states. He Mea Ono brand poi contained 45 total analytes, 8 of which were found only in the “fresh” state, 9 of which were found only in the “aged” state, and 28 of which were found in both the fresh and aged states. Taro brand poi contained 36 total analytes, 7 of which were found only in the “fresh” state, 13 of which were found only in the “aged” state, and 16 of which were found in both the fresh and aged states.

**Figure 1:**
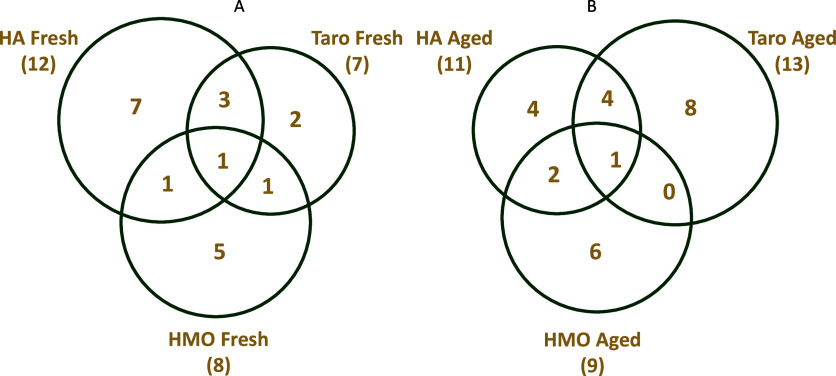
Venn diagram illustrating the relationship between number of compounds found in Hanalei (HA) brand poi, He Mea Ono (HMO) brand poi, and Taro brand poi for (A) fresh samples and (B) aged samples. Numbers in each space represent the number of analytes identified in common between groups. Numbers in parentheses for each brand provide the total number of analytes detected for the indicated aging state.

Looking beyond the core profile, comparing each brand in the “fresh” state only (excluding components shared between fresh and aged states) illustrated the initial differences and similarities among the different commercial products. The analyte shared among all three brands was 4,4-dimethoxy-3-methylbutan-2-one. Hanalei and He Mea Ono fresh shared penta-1,4-diene, Hanalei and Taro fresh shared butane-2,3-dione, 2-methylpropanal, and 2,2,3,3-tetramethyloxirane, while He Mea Ono and Taro fresh shared 1-hydroxypropan-2-one (hydroxyacetoin). The analytes unique to each brand in the “fresh” state are listed in Table 2. Considering analytes only found in the “fresh” state, each brand of poi contains a few more unique VOCs than shared ones, which indicates a relatively distinct flavor profile for each brand when it is “fresh”.

**Table 2:** List of Components Unique to Hanalei Brand Poi, He Mea Ono Brand Poi, and Taro Brand Poi in the “Fresh” State Only

Hanalei fresh	He Mea Ono fresh	taro fresh
2-methylbutanal	(methyldisulfanyl)methane	2-hydroxypentan-3-one
3,5-dimethylheptane	2-methylthiolan-3-one	butyl acetate
3-methylbut-3-enyl acetate	benzene	
acetaldehyde	heptyl acetate	
ethyl propanoate	hexyl acetate	
methyl 3-methyl-2-oxobutanoate		
pyridine		

Comparing each brand in the “aged” state illustrates only the similarities and differences between each brand after fermentation had occurred. All three brands shared one common analyte when aged, 2,5-dimethylfuran, resulting from the degradation of carbohydrates during fermentation. Overall, as aging occurred, Taro brand poi became more similar to Hanalei brand poi and less similar to He Mea Ono brand poi. Similarly, the He Mea Ono brand poi became more like Hanalei brand poi. However, Taro and He Mea Ono remained relatively dissimilar and did not share any components other than 2,5-dimethylfuran. Taro shared 4 analytes with Hanalei in the aged state: 1-butanol, 3-methylbutan-2-ol, cyclopentene, and formyl acetate. Aged Hanalei shared two components with He Mea Ono, butanoic acid and urea. The analytes unique to each brand in the “aged” state are listed in Table 3.

**Table 3:** List of Components Unique to Hanalei Brand Poi, He Mea Ono Brand Poi, and Taro Brand Poi in the “Aged” State Only

Hanalei aged	He Mea Ono aged	taro aged
2-aminopropanoic acid	2-methylbutanal	1-prop-2-ynoxypropan-2-ol
2-methylthiolan-3-one	3-methyl-1-phenylmethoxybut-3-en-2-ol	2-methylpropyl acetate
3-amino-2-hydroxypropanoic acid	3-methylbutanal	3-methylbutyl acetate
pentanoic acid	3-methylbutyl propanoate	ethyl acetate
	3-methylhept-1-ene	ethyl butanoate
	5-methylhex-1-ene	methyl acetate
		n-propyl acetate
		penta-1,4-diene

The above comparisons were based largely on qualitative data about the presence or absence. However, in order to confirm compound presence, abundance data needed to be compared between fresh samples and aged samples as well as analytical controls such as water and instrument blanks. The presence of a compound in each brand was confirmed by examining box and whisker plots, which demonstrate relative quantities of detected analytes as shown in Figure 2. For example, the compound 2,5-dimethylfuran was found in all three brands in only the “aged” state, although each brand contained different amounts of the analyte. Figure 2 demonstrates the method of ensuring higher certainty in Tables 2 and 3 compound lists.

**Figure 2:**
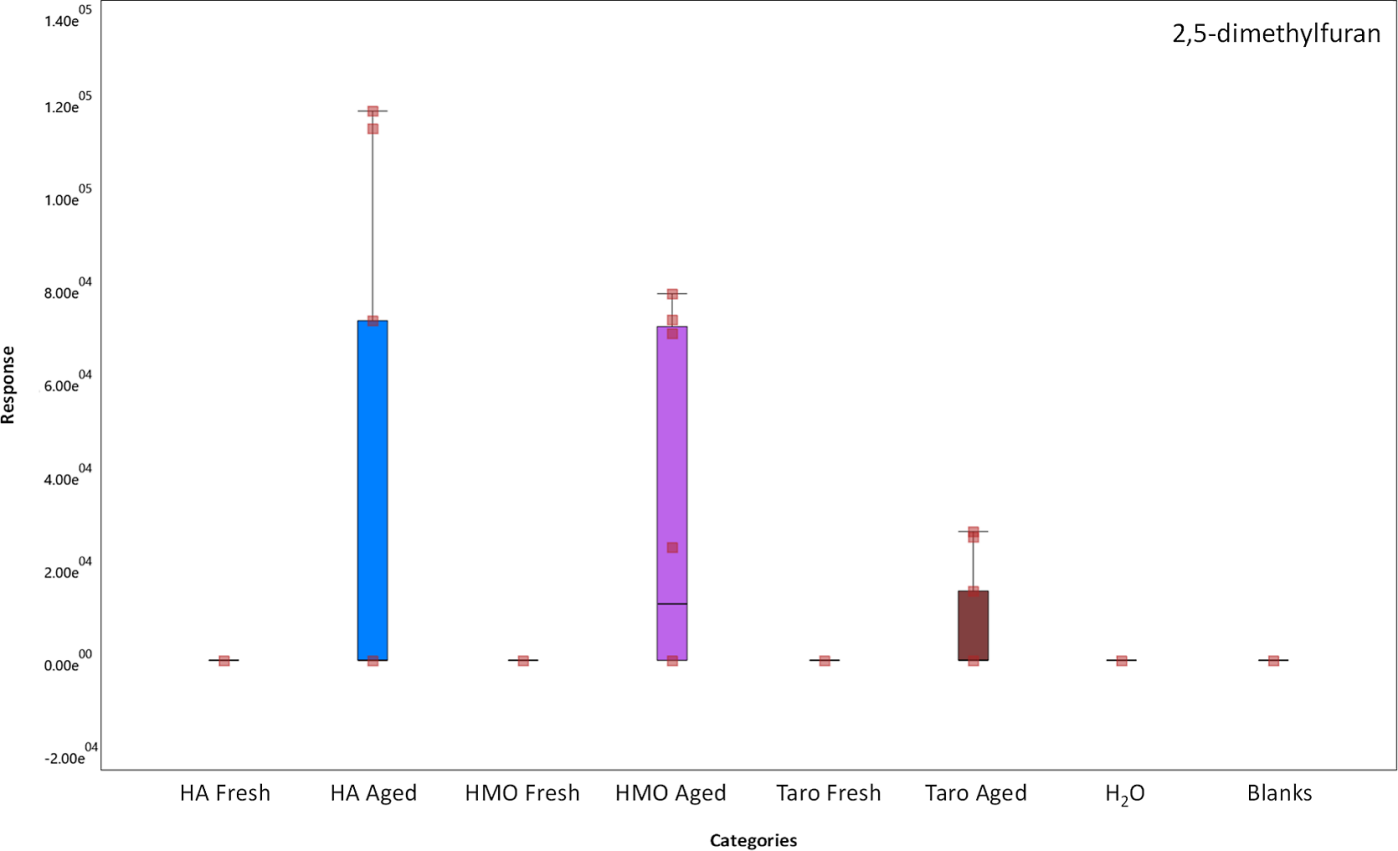
Box and Whisker plot depicting the presence of 2,5-dimethylfuran across all brands in the aged and fresh states, as well as in blanks and H_2_O. Hanalei brand poi is referred to as “HA”, He Mea Ono brand poi is referred to as “HMO”, and Taro brand poi is referred to as “Taro”.

### Changes in the VOC Profile of Poi Products as Fermentation Occurs

Examining the trends in compound classes as commercial poi products ferment provides information about the changes in the VOC profile that occur during their fermentation. Not accounting for aging status, the 56 total analytes were spread between 11 compound classes with esters contributing to 33.93% of the total volatile profile, alcohols contributing to 12.50%, ketones, nitrogen-containing compounds, and alkenes contributing 8.93% each, aldehydes and carboxylic acids both contributing 7.14% each, sulfur-containing compounds, heterocyclic compounds, and alkanes accounting for 3.57% each, and epoxides making up 1.79%.

Comparing the compound classes of the analytes found in only fresh states and the analytes found in only aged states reveals a shift in the number of components in each class as aging occurs (Figure 3). While changes differed by brand, during aging, the number of alcohols and carboxylic acids increased across all the brands and the number of ketones decreased across all the brands, both changes consistent with the fermentation process. Taro brand poi saw an increase in esters and a decrease in aldehydes. In Hanalei brand poi and He Mea Ono brand poi, the number of esters decreased during aging. However, Hanalei brand poi had a decrease in aldehydes during aging while He Mea Ono brand poi had an increase in aldehydes.

**Figure 3:**
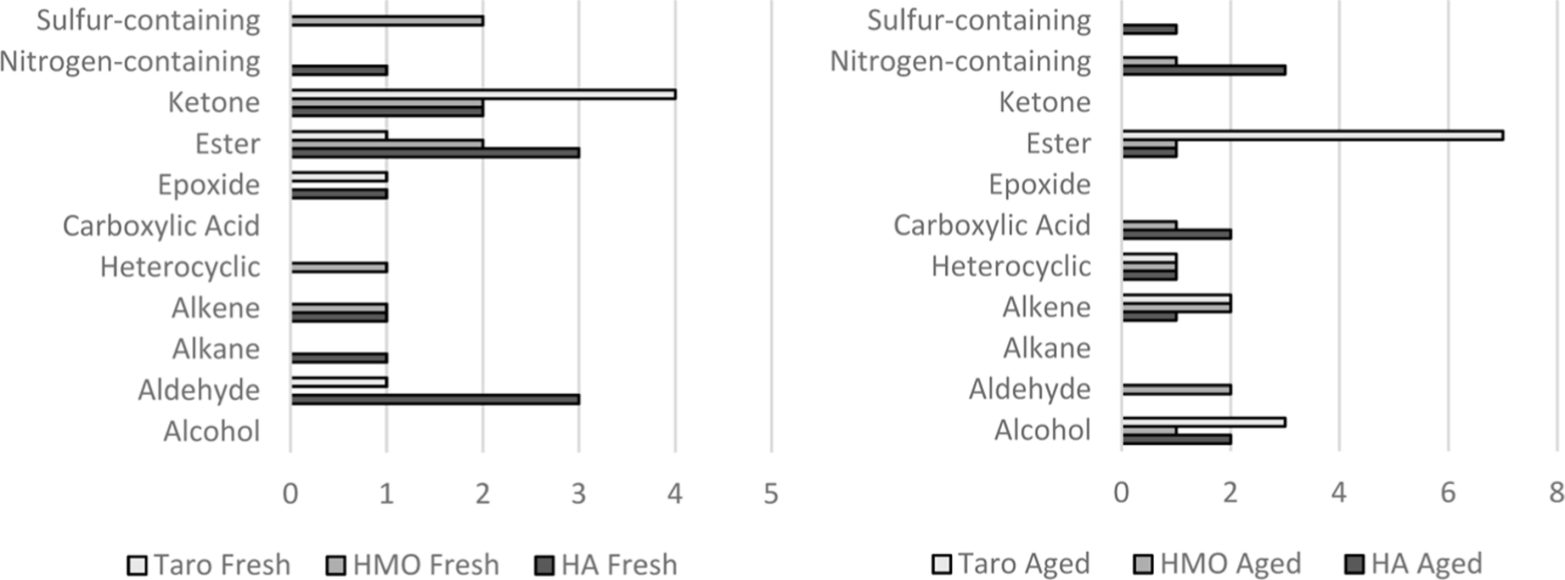
Bar charts demonstrating the number of analytes (count) identified for each compound class among analytes found only in the fresh state across Hanalei (HA) brand poi, He Mea Ono (HMO) brand poi, and Taro brand poi (left) and compound classes among analytes found only in the aged state across Hanalei (HA) brand poi, He Mea Ono (HMO) brand poi, and Taro brand poi (right). Error bars are not shown because total count was used.

The 24 core components previously identified belong to the same compound classes across the fresh and aged states due to their consistent presence in both aging states, so there was no shift seen when analyzing the fresh and aged states separately. Out of the 24 core components, esters contributed to 33.33%, alcohols and ketones contributed to 12.50% each, aldehydes, ketones, and nitrogen-containing compounds accounted for 8.33% each, and alkanes, alkenes, heterocyclic compounds, and epoxides made up 4.14% each. The distribution of class compounds within the core components is similar to the overall distribution of class compounds when looking at all 54 analytes. However, slight differences between the two distributions suggest that changes did occur during fermentation.

Data presented above in Figures 1–3 are based on the compound presence and number of compounds identified. Another method of visualizing the changes that occurred to the VOC profile of commercial poi products during fermentation is Principal Component Analysis (PCA) based on peak areas for each analyte, as shown in Figure 4. A PCA scores plot demonstrates similarities between different samples based on the entire multivariate structure of a data set, and 95% confidence ellipses represent the confidence interval around a defined group. The method and water blanks were included in the PCA to provide a point of comparison for which poi samples exhibited the least abundant VOC profile. Water and blank samples exhibited very low abundance for all VOCs detected, as noted in Figure 2; therefore, the sample groupings located close to the water and blank samples had a lower abundance of VOCs in the samples.

**Figure 4:**
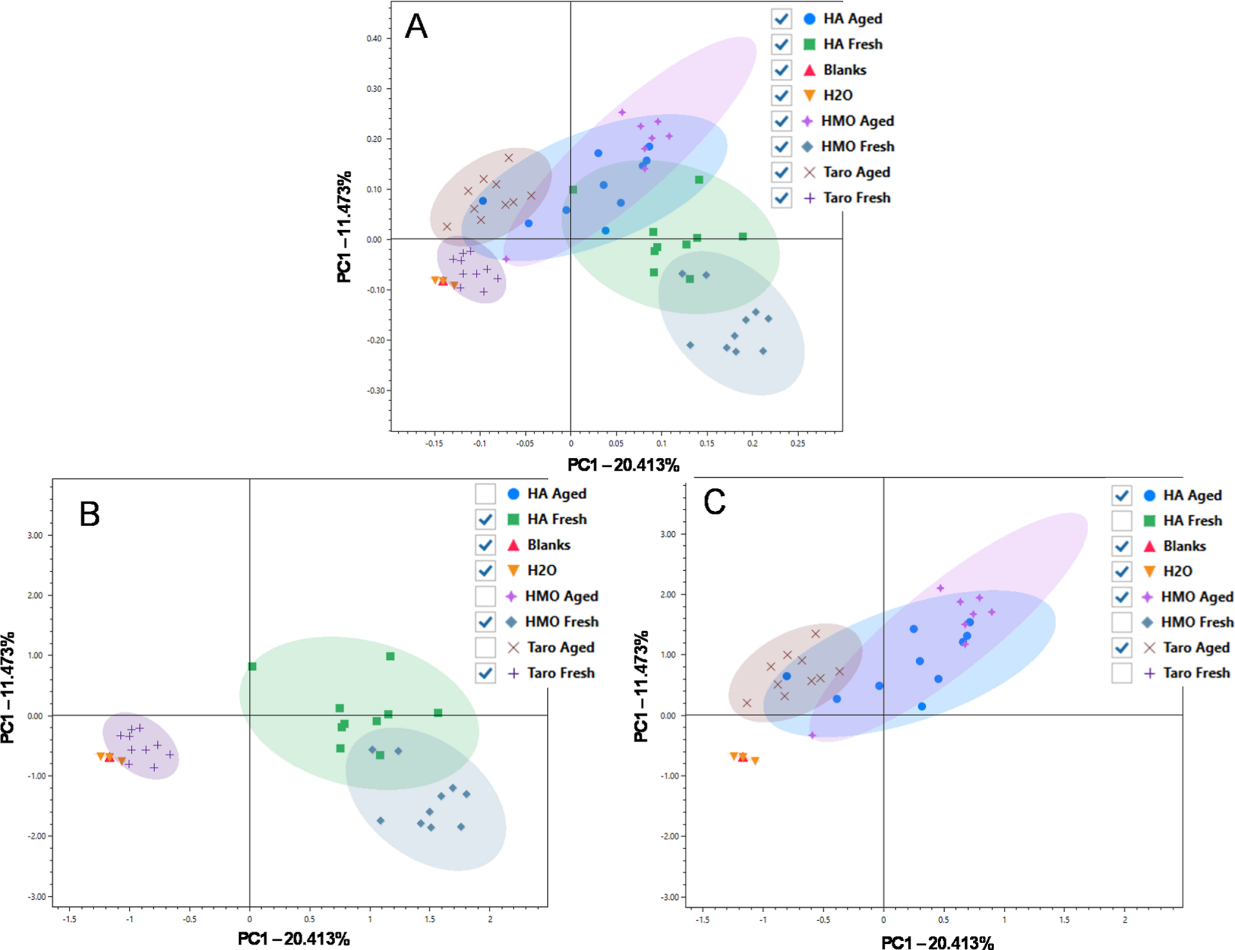
PCA scores plot depicting (A) all fresh samples, all aged samples, water, and blanks, (B) “Fresh” PCA scores plot, (C) “Aged” PCA scores plot. Category marker key pictured far right. Hanalei brand poi is referred to as “HA”, He Mea Ono brand poi is referred to as “HMO”, and Taro brand poi is referred to as “Taro”. Colored circles represent pareto scaling with Log transformations and a 95% confidence ellipse around the different groups. Note that all fresh and aged samples are plotted in the same PCA scores plot, and plots (A) and (B) were generated by hiding or viewing groups within software. They were not generated based on two separate analyses but are shown on two different plots to improve visualization of fresh and aged states.

For each brand, there was an upward shift along the second principal component between the fresh (Figure 4B) and aged (Figure 4C) versions of the samples, which demonstrates the change in the composition due to fermentation. Comparing the brands in the fresh state reveals overlap between the composition of Hanalei and He Mea Ono, with no overlap between Taro and the other brands. Taro overlaps with the water and blanks, signifying that the brand had a low concentration of volatiles present relative to the other brands despite the fact that it was diluted with less water than the other diluted brand, He Mea Ono. Taro brand poi had approximately 10 less volatile substituents than Hanalei brand poi or He Mea Ono brand poi, further accounting for the differences. Moreover, the samples plotted within the confidence ellipse for Taro are closer together than the samples plotted for Hanalei or He Mea Ono brand poi indicating that the compounds detected within Taro brand poi exhibited less variation than the compounds detected within either Hanalei brand poi or He Mea Ono brand poi. Individual analyses on only fresh and only aged samples were also run separately (Supporting Information Figures S4 and S5). These plots also confirmed the trends observed in Figure 4B,C.

Comparing the brands in the aged state shows overlap between all three brands, with Hanalei brand poi sharing similarities with both Taro and He Mea Ono brand poi. There is limited overlap between Taro in the aged state and He Mea Ono in the aged state, which is consistent with the findings from cross-brand comparisons.

The shift in the VOC profile during fermentation results in different aromatic profiles for each commercial brand of poi in fresh and aged state. Based on the VOCs identified in each brand, odor descriptors were assigned to each brand in the “fresh” and “aged” states in Table 4.

**Table 4:** Aroma Descriptors Highlighting the Differences in the Flavor Profile of Fresh and Aged Samples Across Hanalei Brand Poi, He Mea Ono Brand Poi, and Taro Brand Poi

	Hanalei	He Mea Ono	taro
fresh	fruity	fruity	fruity
	nutty	floral	nutty
	floral	green	caramel
	sweet	savory	dairy
	earthy	sweet	floral
	creamy	earthy	green
	vegetal	creamy	savory
	cocoa	caramel	earthy
			
aged	fruity	fruity	fruity
	nutty	floral	floral
	floral	nutty	sweet
	sweet	sweet	nutty
	savory	dairy	creamy
	creamy	alcoholic	malt
	tangy	sour	acidic
	sour	acidic	alcoholic

The aroma descriptors in Table 4 were derived from compiling the list of VOCs in each brand in the fresh state and aged state and categorizing the common odors. For example, the VOC 3-methylbutyl acetate, which was found in Hanalei brand poi, He Mea Ono brand poi, and Taro brand poi in various aging states, has flavor descriptors apple, banana, and pear^19^ which are grouped in a “fruity” category. The same method was applied to each VOC present in the Hanalei brand poi, He Mea Ono brand poi, and Taro brand poi.

An overall shift in the aromatic descriptors occurred during fermentation. While the predominant descriptors in both the “fresh” and “aged” state included fruity, floral, earthy, and sweet, the “aged” state also developed tangy, sour, acidic, and alcoholic notes. As the number of alcohols and carboxylic acids increased with aging across all brands, this shift in the aromatic profile was expected.

The shift in the odor profile was of interest due to the impact that flavor has on consumer consumption. Poi, as a naturally probiotic food, could potentially be useful for digestive disorders such as gastroenteritis and irritable bowel syndrome;^3^ therefore, consumer perception of its aromas is essential in determining the extent of its consumption. Chemical profiling of the odors of various brands of poi could enhance the experience of consumers, helping them make more informed decisions based on individual preferences.

Traditionally, poi has been fed to infants with food allergies and patients with stomach ulcers and other nutritional disorders.^1,2^ The potential of poi for addressing gastrointestinal issues is largely due to the antioxidant, antimicrobial, and anti-inflammatory properties documented in C. esculenta.^22,23^ Seven components identified in this study have documented antimicrobial, antifungal, and anti-inflammatory properties. Acetaldehyde, 3-hydroxybutan-2-one, butane-2,3-dione, acetic acid, and pentanoic acid all demonstrated antimicrobial character, inhibiting the growth of pathogenic bacteria in the gut.^24–26^ 2,5-dimethylfuran showed antifungal activity and was demonstrated to suppress the growth of pathogenic fungi.^27^ Butanoic acid is widely established as an antimicrobial and anti-inflammatory agent.^25,26^ Studies have linked a lack of butyrate-producing bacteria to ulcerative colitis and other emerging diseases.^28^ All brands of poi analyzed in this study contained a similar number of these antimicrobial, anti-inflammatory compounds (Table 5).

**Table 5:** List of Analytes with Documented Antimicrobial and/or Anti-inflammatory Properties and Their Presence in Hanalei (HA) Brand Poi, He Mea Ono (HMO) Brand Poi, and Taro Brand Poi in the Fresh and Aged States

components	HA fresh	HA aged	HMO fresh	HMO aged	taro fresh	taro aged
acetaldehyde	x		x	x	x	x
2,5-dimethylfuran		x		x		x
butanoic acid		x		x		
acetic acid	x	x	x	x	x	x
pentanoic acid		x				
3-hydroxybutan-2-one	x	x	x	x	x	x
butane-2,3-dione	x				x	

The VOC profile identified for each brand of poi not only contributes to the odor and consumer perception of the product but also can serve as important identifiers for the medicinal use of poi. In this way, chemical profiling of poi and other fermented food products can offer insight into their historical use and future potential as moderators of gut health. The specific use of GC×GC could also be expanded to the profiling of other fermented foods, especially those of cultural significance in areas of the world where food products are viewed as traditional medicines, to improve understanding of how VOCs impact the nutritional and medicinal value and perception of products. It is also possible that GC×GC methods for food profiling could be used in monitoring the quality of products such as poi (or others) to investigate core profiles, differences between brands, and manufacturing processes, as well as best practices for food storage.

## Conclusions

The goal of this study was 3-fold in establishing a core VOC profile of poi, differences in VOC profiles of three commercial poi products, and changes to the VOC profile that occur during the fermentation of poi. The core VOC profile of poi consisted of 24 VOCs spanning 10 different compound classes contributing to a general sweet, fruity, and nutty aroma. The three brands examined, Hanalei brand poi, He Mea Ono brand poi, and Taro brand poi, each had a distinct aromatic profile in the “fresh” state versus the “aged” state. While the brands overlapped in a number of VOCs, unique components help to differentiate each brand from the others. The overall chemical shift from “fresh” to “aged” involved a difference in the number of analytes present in each compound class. Alcohols and carboxylic acids increased consistently with aging among all brands, while ketones decreased with aging among all brands. The use of PCA established the similarities between brands based on component peak areas. Further studies could explore the odor profile of poi at different stages in the aging process to determine an “optimal” time of consumption based on chemical data.

This study is significant in its exploration of the VOC profile of poi in connection with its traditional medicinal uses and its contemporary place in the consumer market. Applying knowledge about the chemical composition of poi along with its established odor descriptors can explain consumer preferences as well as contribute to the ongoing research about poi’s nutritional and probiotic benefits.

## Methods

### Sample Preparation

Three brands of commercially processed poi were purchased. Brands included Hanalei brand poi, He Mea Ono brand poi, and Taro brand poi. Working poi samples were prepared for each brand according to package instructions the day after purchase. Hanalei and Taro brand poi were mixed with bottled water at dilution ratios of poi to water of 4:1 and 2:1, respectively. He Mea Ono brand poi was not diluted based on package suggestions. For each brand, 10 replicates of approximately 5.00 g of working poi samples were weighed and recorded, transferred into 20 mL headspace vials, and sealed. All 10 replicates were aliquoted from the same commercial package of poi. Each vial was labeled with the brand, replicate number, sample mass, and preparation date. In addition, for each brand, three blank vials and three bottled water samples were prepared as controls. The samples were analyzed in a fresh state shortly after purchase, and the same prepared sample vials were then analyzed again after sitting sealed at room temperature in a cupboard for 7 days.

### SPME Arrow Sampling

Solid phase microextraction arrow sampling with a 1.50 mm wide sleeve divinylbenzene/carbon wide range (CWR) fiber (Restek Corporation, Bellefonte, PA) was performed using 20 mL headspace vials (Restek Corporation) containing approximately 5 g of poi or the extracted water sample. This fiber was chosen due to success in similar studies.^9^ The exact mass of each sample was recorded on an analytical balance for normalization purposes in data processing. Sample extraction and injection were performed using a TriPlus RSH Autosampler (Thermo Scientific, Waltham, MA, USA). Sample incubation was performed at 35 °C for 5 min at 500 rpm. Sample extraction was performed at 35 °C for 5 min at 1000 rpm. The needle speed was 20 mm/s in the vial, and the needle depth was set to standard. The incubation mode was constant. Injection was performed to a depth of 45 mm with a 35 mm/s penetration speed for 1 min. SPME arrow fibers were conditioned for 30 min at 260 °C prior to each sequence and were confirmed as blank with a fiber blank injection. SPME arrow fibers were reconditioned with a predesorb time of 2 min and a postdesorb time of 5 min between each run. All samples were run over a short period (less than 3 days) for each condition (fresh and aged), and no other projects were run between the two analyses.

### GC×GC–qMS Method

The instrument used for analysis of VOCs from poi samples was a TRACE1300 GC/FID and an ISQ 7000 Single Quadrupole Mass Spectrometer (Thermo Scientific). Although FID data were collected at the time of sample analysis as part of a course-based undergraduate research experience, the FID data were not included in the analysis, and only the qMS data were used herein. The inlet was operated in split mode with a split flow of 20 mL/min and a purge flow of 2.000 mL/min. The inlet temperature was 250 °C. The first-dimension column was an Rxi-624Sil MS column (30 m × 0.25 mm ID × 1.4 μm film thickness, Restek Corporation), and the second-dimension column was a Stabilwax (5 m × 0.25 mm × 0.25 μm film thickness, Restek Corporation). An INSIGHT Reverse Fill/Flush Modulator (SepSolve Analytical, Peterborough, UK) was used with a modulation period of 2.5 s and a flush time of 100 ms. The loop dimensions were 0.53 × 1133 mm ID with a resulting loop volume of 25 μL. The bleed line dimensions were 5 × 0.1 mm ID. Ultrahigh purity helium (Airgas, Radnor, PA, USA) was used as the carrier gas. The flow rate in the first-dimension column was 1.00 mL/min, and the auxiliary gas flow rate was 20.0 mL/min, with a calculated flow rate of 17.9 mL/min in the second dimension. The flow was split between the FID and MS using an unpurged SilFlow GC 3-port splitter (Trajan Scientific and Medical, UK) at approximately 4.5:1, which was maintained throughout the run. An uncoated fused silica column was used to connect the splitter device to each of the FID and MS. The GC oven started at 50 °C, was held for 1 min, increased to a final temperature of 250 °C at the rate of 5 °C/min, and held for 1 min. The total run time was 42 min.

The ion source temperature and the transfer line temperature for the qMS were both set to 280 °C. The qMS was operated in the electron ionization mode with a scan range of 40 to 300 *m/z*. The total scan time was 0.0241 s, which resulted in an acquisition rate of 41.5 scans/s. This is the maximum acquisition rate for this instrument when using this scan range. Instrument control was performed using Chromeleon 7 version 7.2.9 (Thermo Scientific).

### Data Processing Method

All sample files were imported into AnalyzerPro XD (ver. 1.15.8853.26842, SpectralWorks Ltd.) into the sequence window. Samples were categorized by brand (Hanalei, Taro, and He Mea Ono) and aging status (Fresh or Aged), with additional categories for blanks and water. A 2D processing method was applied with modulation frequency of 2.5 s, modulation window of 40%, matching of 50%, minimum of three, and maximum of 200. Library searching was set to both 1D and 2D, and target analysis was set to 1D only. Analytes were processed as components with a minimum number of four masses. The mass range was processed from 40 to 300 *m/z*, and the retention time was processed as range of 6 to 40 min. The area threshold for detection was set to 500, the height threshold was zero, and the scan window and signal-to-noise were set to three. Three smoothing points were used: width was 0.002 min, temporal resolution was minimum, fronting was 0%, and tailing was 2%. Library searching was enabled using mainlib and replib from The National Institute of Standards and Technology (NIST) 2023 Mass Spectral Library. 10 library hits were returned with forward and reverse thresholds of 700. The confidence threshold was 65% with a confidence ratio of 70:30. Targets were matched using a retention time with a 0.2 min retention time window. No standards were used in confirming analyte identification; therefore, compound identifications should be considered tentative.

Within the sequence results, groupings were toggled to view category comparisons. Visualization included PCA, box and whisker plots, and bar charts, all generated within AnalyzerProXD. Group categories used included: Hanalei (HA) aged and fresh, He Mea Ono (HMO) aged and fresh, Taro aged and fresh, Water, and Blanks. Peak areas were normalized based on sample weight in software during initial processing and for PCA, box and whisker plot visualization, and bar charts. In order to filter compounds out of the analysis that were found infrequently in a group, a threshold of 30% detection within class was used, leaving only compounds on the list which appeared in greater than 30% of the samples within each group (i.e., at least 3 of 10 replicates). This additionally facilitated the calculation of average and % relative standard deviation (% RSD) for each compound and decreased the matrix sparsity for PCA. Components that were found sporadically (i.e., found in one or two replicates in a single brand) were removed via this 30% detection filter, and additional compounds not logical to occur in natural products were manually eliminated from the final analysis (i.e., plasticizers, solvent artifacts, column bleed).

## Supplementary Material



## Data Availability

The data are available from the corresponding author upon reasonable request.

## Notes

Additional peak table information, chromatograms, and plots resulting from PCA (PDF)
