# Supplementary material for: Volatile Organic Compound Profiling of Commercial Poi Products in Fresh and Aged States Using Comprehensive Two-Dimensional Gas Chromatography
Source: ACS Omega. 2025 May 15;10(20):20754–62. doi: 10.1021/acsomega.5c01820 (PMC12120601; doi:10.1021/acsomega.5c01820)

Volatile Organic Compound Profiling of Commercial Poi Products in Fresh and Aged States Using Comprehensive Two-Dimensional Gas Chromatography

Sarah C. Foster[1], Cynthia Cheung[2], Laura Tipton[3,4], Jonathan D. Baker[3], Kahoalii K. Keahi-Wood[3], Katelynn A. Perrault Uptmor*[1,2]

[1] Nontargeted Separations Laboratory, Chemistry Department, William & Mary

[2] Laboratory of Forensic and Bioanalytical Chemistry/Forensic Sciences Unit, Chaminade University of Honolulu

[3] School of Natural Sciences and Mathematics, Chaminade University of Honolulu

[4] Departments of Biology and Mathematics & Statistics, James Madison University

***Corresponding Author:**

Katelynn A. Perrault Uptmor
Nontargeted Separations Laboratory
Chemistry Department
William & Mary
540 Landrum Drive
Williamsburg, VA 23185

Table S1. Complete list of analytes identified in three brands of commercial poi, sorted by brand, aging status, and compound classification. "HA" refers to Hanalei brand poi, "HMO" refers to He Mea Ono brand poi, and "Taro" refers to Taro brand poi. Aroma descriptors provided based on available information in PubChem and safety data sheets (SDS) as mentioned in the body of the text.

Components	HA Fresh	HA Aged	HMO Fresh	HMO Aged	Taro Fresh	Taro Aged	Class	Aroma Descriptors
1-butanol		x				x	alcohol	fruit
1-pentanol	x	x	x	x	x	x	alcohol	balsamic fruit green pungent yeast
1-prop-2-ynoxypropan-2-ol	x	x	x	x		x	alcohol	
2-methylpropan-1-ol	x	x	x	x	x	x	alcohol	apple bitter cocoa wine
3-methyl-1-phenylmethoxybut-3-en-2-ol				x	x	x	alcohol	
3-methylbutan-2-ol		x				x	alcohol	green
butane-2,3-diol			x	x			alcohol	fruit onion
2-methylbutanal	x			x	x	x	aldehyde	almond cocoa fermented hazelnut malt
2-methylpropanal	x				x		aldehyde	burnt caramel cocoa green malt
3-methylbutanal				x	x	x	aldehyde	cocoa almond malt
acetaldehyde	x		x	x	x	x	aldehyde	floral green apple
3,5-dimethylheptane	x						alkane	
pentane	x	x	x	x	x	x	alkane	
3-methylhept-1-ene				x	x	x	alkene	
5-methylhept-1-ene	x	x					alkene	
5-methylhex-1-ene				x	x	x	alkene	
cyclopentene		x				x	alkene	
penta-1,4-diene	x		x			x	alkene	
2,5-dimethylfuran		x		x		x	heteroaroma tic	savory
benzene			x				aromatic hydrocarbon	
butanoic acid		x		x			carboxylic acid	butter cheese sour
acetic acid	x	x	x	x	x	x	carboxylic acid	acid fruit sour

								pungent vinegar
pentanoic acid		x					carboxylic acid	cheese pungent
propanedioic acid	x	x	x	x	x	x	carboxylic acid	sweet earth
2,2,3,3-tetramethyloxirane	x		x	x	x		epoxide	
2-methylpropyl acetate	x	x	x	x		x	carboxylic acid ester	apple banana floral herb
2-phenylethyl acetate			x	x			ester	
3-methylbut-3-enyl acetate	x		x	x			carboxylic acid ester	fruit
3-methylbutyl acetate	x	x	x	x		x	ester	apple banana bear
3-methylbutyl propanoate				x			carboxylic acid ester	apple apricot pineapple
3-oxobutan-2-yl acetate			x	x			carboxylic acid ester	
butyl acetate	x	x	x	x	x		carboxylic acid ester	apple banana pungent
ethyl 2-methylpropanoate	x	x	x	x			carboxylic acid ester	floral nutty
ethyl acetate	x	x	x	x		x	carboxylic acid ester	aromatic brandy grape
ethyl butanoate	x	x				x	carboxylic acid ester	apple butter cheese pineapple strawberry
ethyl propanoate	x		x	x	x	x	carboxylic acid ester	apple pineapple rum strawberry
formyl acetate		x	x	x		x	carboxylic acid ester	
heptyl acetate	x	x	x				carboxylic acid ester	floral fresh
hexyl acetate	x	x	x				carboxylic acid ester	apple banana grass herb pear
methyl 3-methyl-2-oxobutanoate	x		x	x			keto ester	
methyl acetate	x	x	x	x		x	ester	ester green
n-propyl acetate	x	x	x	x		x	ester	celery floral pear red fruit
pent-4-enyl acetate	x	x	x	x			ester	green
pentyl acetate	x	x	x	x			ester	sweet fruit

1-hydroxypropan-2-one			x		x		ketone	butter herb malt pungent
2-hydroxypentan-3-one	x	x	x	x	x		ketone	earth nutty
3-hydroxybutan-2-one	x	x	x	x	x	x	ketone	butter creamy green pepper
4,4-dimethoxy-3-methylbutan-2-one	x		x		x		ketone	
butane-2,3-dione	x				x		ketone	butter pastry yeast
2-aminopropanoic acid		x			x	x	nitrogen-containing	
3-amino-2-hydroxypropanoic acid		x					nitrogen-containing	
aminourea	x	x	x	x	x	x	nitrogen-containing	
pyridine	x		x	x			nitrogen-containing	
urea		x		x	x	x	nitrogen-containing	
(methyldisulfanyl)methane	x	x	x				sulfur-containing	cabbage garlic onion
2-methylthiolan-3-one		x	x				sulfur-containing	cabbage must onion

Figure S1. Total ion current chromatograms comparing the fresh states for each poi brand investigated. Plots were produced using GC×GC-qMS data.

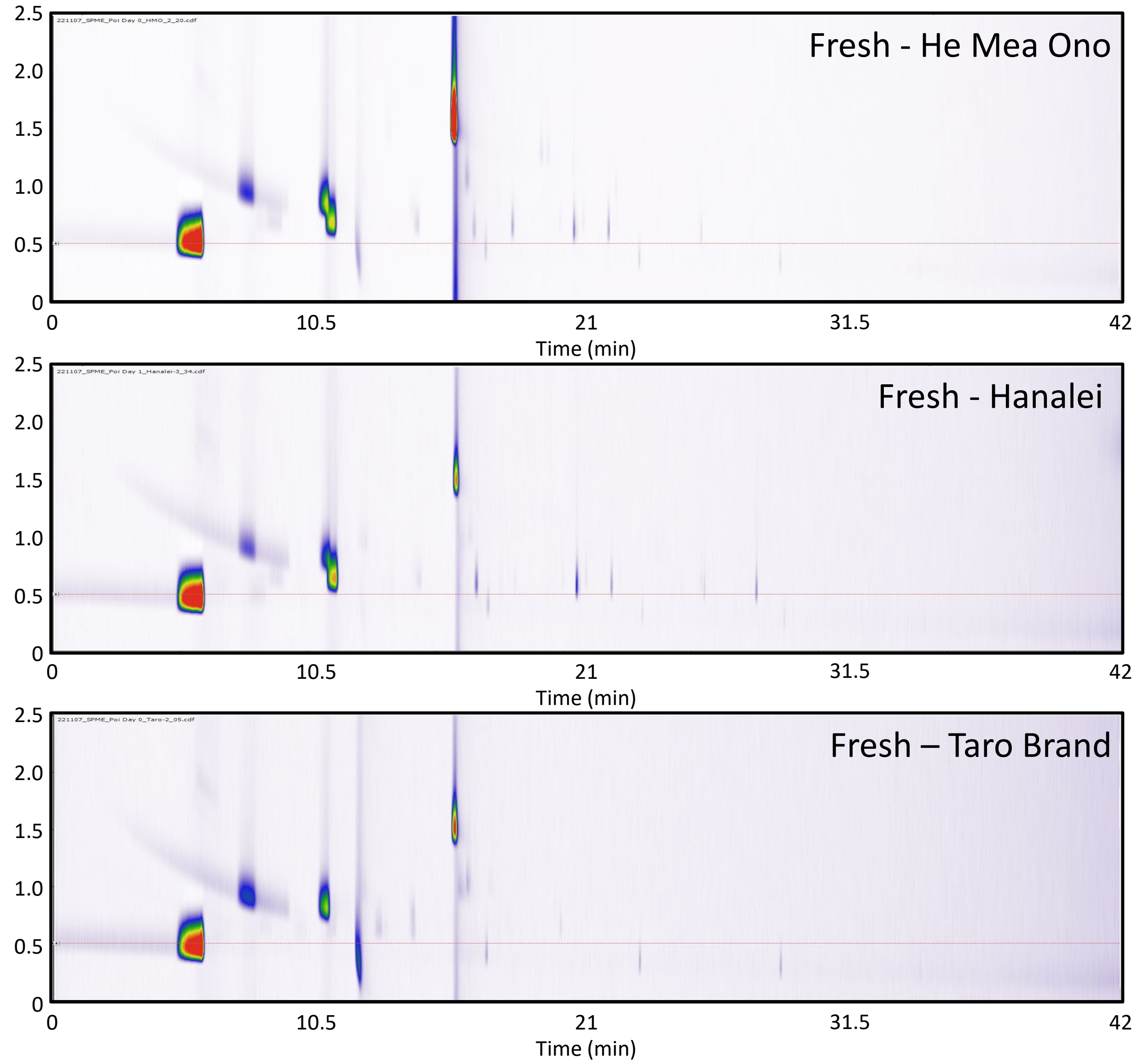

Figure S2. Total ion current chromatograms comparing the fresh states for each poi brand investigated. Plots were produced using GC×GC-qMS data.

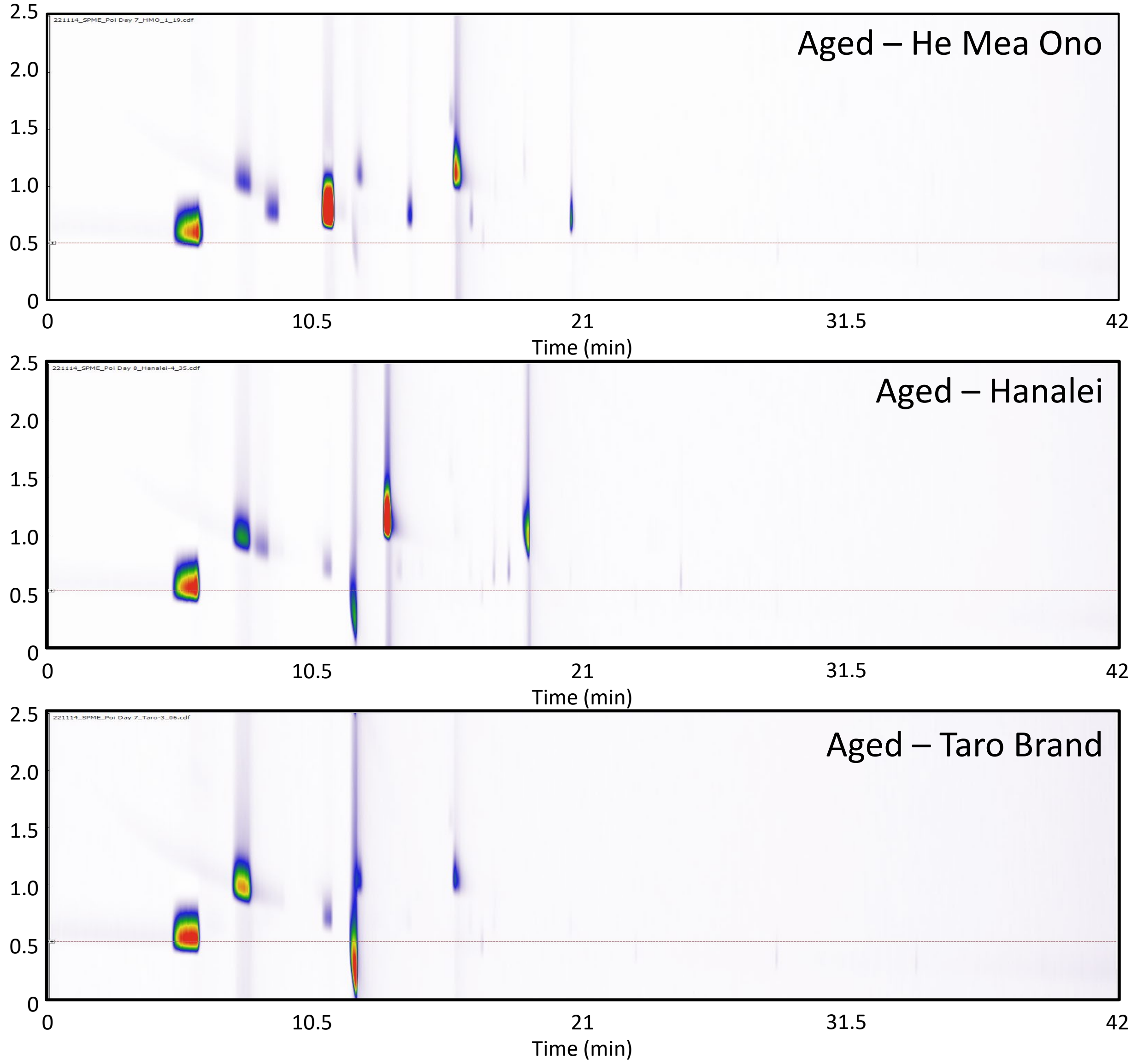

Figure S3. Principal Component Analysis (PCA) loadings plot for GC×GC-qMS data from commercially analyzed poi samples corresponding to Figure 4A in the main text.

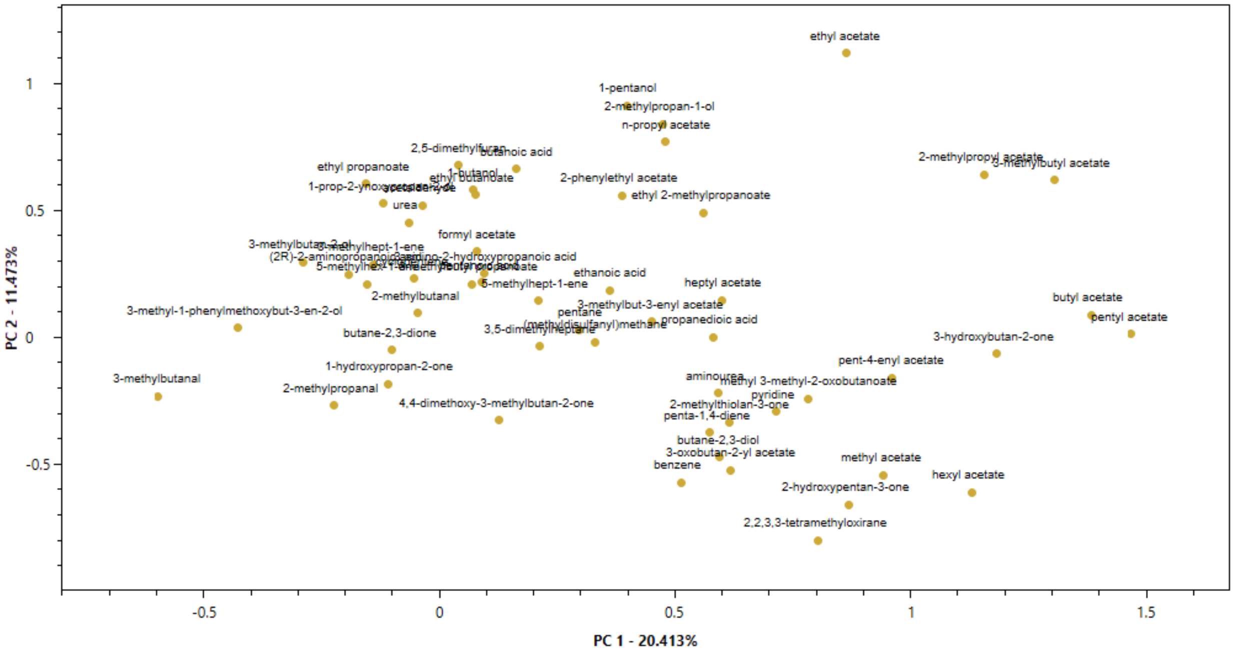

Figure S4. Principal Component Analysis (PCA) scores plot depicting fresh samples with blanks and water samples. Category marker key pictured far right. Hanalei brand poi is referred to as "HA", He Mea Ono brand poi is referred to as "HMO", and Taro brand poi is referred to as "Taro". Colored circles represent pareto scaling with Log transformations and a 95% confidence ellipse around the different group.

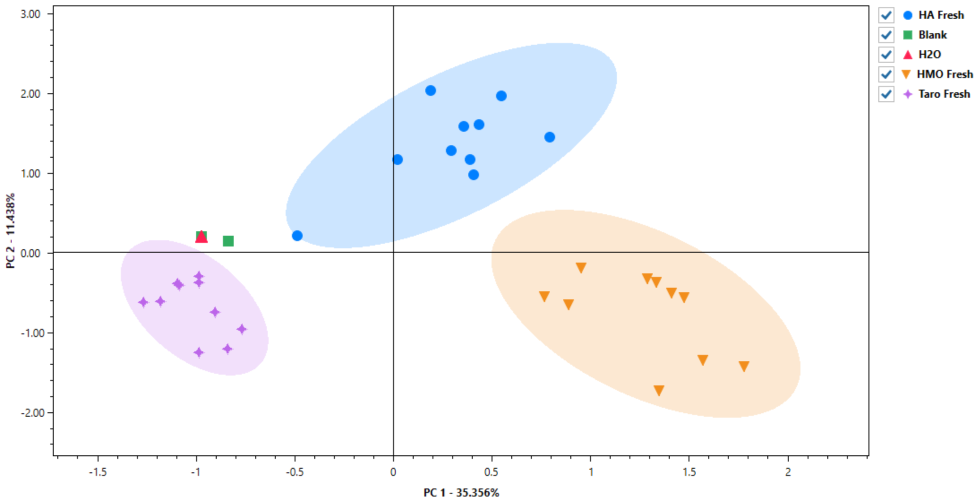

Figure S5. Principal Component Analysis (PCA) scores plot depicting aged samples with blanks and water samples. Category marker key pictured far right. Hanalei brand poi is referred to as "HA", He Mea Ono brand poi is referred to as "HMO", and Taro brand poi is referred to as "Taro". Colored circles represent pareto scaling with Log transformations and a 95% confidence ellipse around the different group.

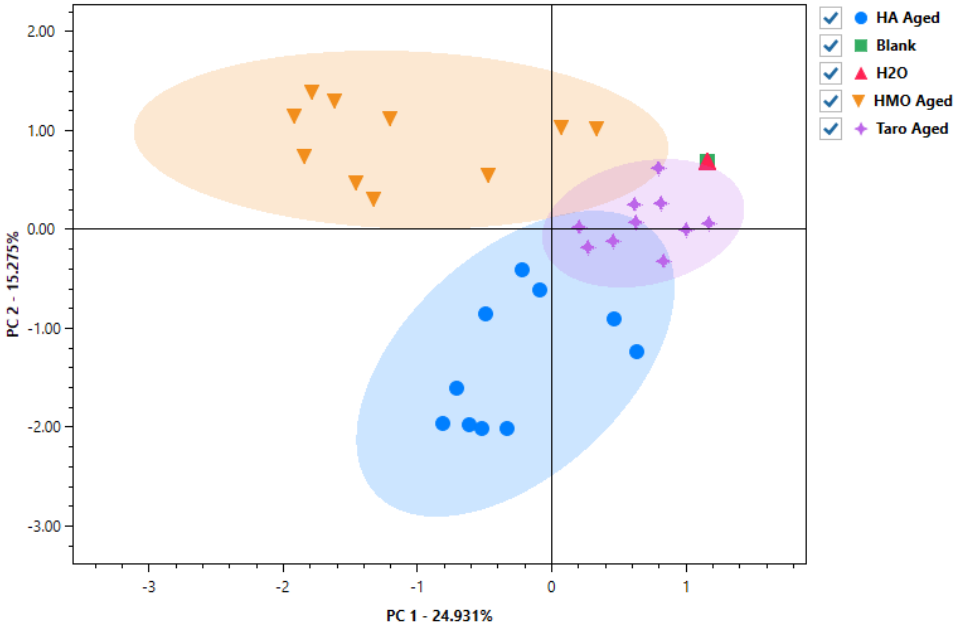

Supplement: Supplementary file 1 [file ao5c01820_si_001.pdf]
